# Astrocytic endfeet re-cover blood vessels after removal by laser ablation

**DOI:** 10.1038/s41598-018-37419-4

**Published:** 2019-02-04

**Authors:** Hideaki Kubotera, Hiroko Ikeshima-Kataoka, Yoshiki Hatashita, Anna Letizia Allegra Mascaro, Francesco Saverio Pavone, Takafumi Inoue

**Affiliations:** 1 Laboratory of Neurophysiology, Department of Life Science and Medical Bioscience, Faculty of Science and Engineering, Waseda University, Tokyo, Japan; 2 European Laboratory for Non-Linear Spectroscopy, University of Florence, Florence, Italy; 3 Neuroscience Institute, National Research Council, Pisa, Italy

## Abstract

The astrocyte, one of the glial cells, plays many functional roles. These include provision of nutrients from blood vessels to neurons, supply of neurotransmitters and support of blood–brain barrier (BBB) integrity. Astrocytes are known to support the integrity of BBB through maintenance of the tight junction between endothelial cells of blood vessels. However, evidence of its direct contribution to BBB is lacking owing to technical limitations. In this study, astrocytic endfeet covering blood vessels were removed by the laser ablation method with two photon laser scanning microscopy in *in vivo* mouse brain, and the re-covering of blood vessels with the astrocytic endfeet was observed in about half of the cases. Blood vessels kept their integrity without astrocytic endfoot covers: leakage of plasma marker dyes, Evans Blue or dextran-conjugated fluorescein, was not observed from stripped blood vessels, while ablation of vascular walls induced extravasation of Evans Blue. These results suggest that the astrocytic endfeet covering blood vessels do not contribute to the immediate BBB barrier.

## Introduction

Astrocytes, a type of central nervous system glial cell, play important roles for maintaining brain homeostasis, such as uptake of glutamate and GABA, provision of nutrients from blood vessels to neurons and control of extracellular pH^1,2^. Further, astrocytes become reactive in response to brain injury and inflammation; reactive astrocytes have different gene expression patterns, roles and morphology from non-reactive astrocytes^3^. The roles of reactive astrocytes include scar formation and preventing the spread of inflammation.

Astrocytes interact with blood vessels with their endfeet. An electron microscopic study indicated that astrocytic endfeet cover almost entire surface of the blood vessels^4^. Astrocytic endfeet play roles in the regulation of dilation and constriction of microvessels to control blood flow^5–7^.

The blood–brain barrier (BBB) is formed by tight junctions among endothelial cells, pericytes and astrocytic endfeet, and restricts entry of neurotoxins and pathogens from the bloodstream into brain parenchyma^8^. BBB malfunction is known to cause neuronal damage, synaptic dysfunction and loss of neuronal connectivity in many neurodegenerative diseases^9^. There are several tight junction proteins expressed between brain endothelial cells such as claudin-5, occludin, ZO-1 and ZO-2, which are essential to maintain BBB integrity^10^. Notably, removal of claudin-5 caused dysfunction of BBB^11^. Pericytes were also shown to be necessary for maintenance of BBB integrity using pericyte-deficient mice^12^.

The astrocytic endfoot is also shown to maintain the BBB. It has been shown to induce the BBB properties of endothelial cells^13^. In studies using co-cultures of astrocytes and endothelial cells, tight junctions between endothelial cells were enhanced in length, width, and complexity^14^, expression of tight junction proteins in endothelial cells were increased, and sucrose permeability was decreased^15^. Our previous studies suggested that activation of astrocytes is essential for recovery of BBB integrity after brain injury^16,17^. In contrast, BBB was not disrupted in astrocyte removal experiments by genetic toxin expression in astrocytes^18,19^.

Although it has been suggested that the astrocytic endfoot is an integral part of BBB and regulates diffusion of solutes and water between blood vessels and brain parenchyma, direct evidence is still lacking. Mice lacking gap junction proteins connexin 43 and 30, which are enriched in the astrocytic endfoot, have dysfunction in BBB^20^. Aquaporin 4 (AQP4, a brain water channel) is expressed in the astrocytic endfoot facing blood vessels and that is suggested to contribute to BBB integrity based on reduced extent of edema in AQP4-deficient mice^21^. However, AQP4-deficient mice did not show BBB malfunction^22^. Thus the endfoot was thought to limit the water diffusion rate between the inside and outside of blood vessels under pathological conditions when water flux is high^4^. Another line of evidence suggests that the astrocytic endfoot could function as a part of molecular sieve of BBB, in which intracellular molecular diffusion in the endfoot was shown to be much slower than in other parts of astrocytes using a caged fluorescent probe^23^. Consequently, there are still debates regarding the astrocytic endfoot’s role in BBB integrity. To understand the functional roles of the astrocytic endfoot in BBB experiments, the removal of not the entire astrocytes or molecules but only the astrocytic endfeet is necessary.

Laser ablation with two-photon laser-scanning microscopy (2PLSM) was adopted for the removal of functions of parts of cells^24^. Irradiation by using a high-energy laser on selected points causes focal damage with a high spatial precision, which can ablate individual dendritic spines of neurons without causing any visible damage to surrounding tissue^25^. This technique has been applied to neurons^26^, microglia^27^ and blood vessels^28^, but not to astrocytes. Furthermore, 2PLSM enables *in vivo* imaging penetrating deep in the tissue with confined photodynamic damage to the vicinity of the focal plane^29^, enabling continuous observation of the same cells over several days^30^.

In this study, we applied the laser ablation technique on astrocytic endfeet and performed *in vivo* imaging of astrocytes and blood vessels with 2PLSM to investigate the functional roles of astrocytic endfeet on the blood vessel. In particular, we focused on changes in the shape of astrocytes after ablating their endfeet and the relationship between astrocytic endfeet and BBB integrity. Transgenic mice expressing enhanced green fluorescent protein (EGFP) in astrocytes driven by the glial fibrillary acidic protein (GFAP) promoter and intraperitoneally (ip) injected Evans Blue (EB) enabled visualization of the shape of astrocytes and blood vessels, respectively. In a subset of experiments, sulforhodamine 101 (SR101) applied to brain surface and ip-injected dextran-conjugated fluorescein isothiocyanate (FITC-dextran) were used for staining astrocytes and blood vessels, respectively. Laser ablation stripped astrocytic endfeet from blood vessels, and the stripped part was re-covered by astrocytic endfeet within a few days. Because there was no leakage of EB or FITC-dextran from the stripped surface of the blood vessels, the endfoot cover of the blood vessel was not considered to be an essential element for the physical barrier of the BBB. Further, this study is the first to demonstrate that the laser ablation technique is applicable to astrocytes.

## Materials and Methods

### Mice

Animal care was performed in accordance with the guidelines outlined by the Institutional Animal Care and Use Committee of Waseda University. The protocol was approved by the Committee on the Ethics of Animal Experiments of Waseda University. Adult (2–8 months old) GFAP-EGFP transgenic mice (Tg(Gfap-EGFP)3739Sart, The Jackson Laboratory, Bar Harbor, ME) were crossed with wild type mice (C57BL/6) to maintain a heterozygous line. The sequences of PCR primers for genotyping were as previously described^31^.

### Operation for cranial window on mouse brain

For *in vivo* imaging and laser ablation, an optical window was created on the skull of mice as described^32,33^. Briefly, mice were anesthetized with an intraperitoneal injection of sodium pentobarbital (65 mg/kg, Kyoritsu Seiyaku, Tokyo, Japan). The skin, muscle and periosteum on the skull were removed, then a metallic head plate (19 mm in length, 12 mm in width and 1 mm in thickness) with a hole (5 mm in diameter) was glued to the skull with dental cement (GC corporation, Tokyo, Japan). Craniotomy (3 mm in diameter) was performed in an area between the coronal and lambdoidal sutures with a dental drill, avoiding the sagittal suture. Dura was removed with a dura hook. The hole in the skull was covered with a circular cover glass (4 mm in diameter, 0.12 mm in thickness, Matsunami, Osaka, Japan) and sealed with cyanoacrylate glue (Aron Alpha, Toagosei CO., LTD., Tokyo, Japan).

### Immunohistochemistry

Mice were anesthetized with isoflurane (DS Pharma Animal Health Co., Ltd., Osaka, Japan) and transcardially perfused with 4% paraformaldehyde (Nacalai Tesque, Kyoto, Japan) in phosphate-buffered saline. Coronal brain sections (100 µm thick) prepared with a vibratome-type tissue slicer (DTK-1000, Dosaka-EM, Kyoto, Japan) were immunostained with rabbit anti-GFAP (1:500, DAKO, Glostrop, Denmark) and rat anti-GFP (1:1000, Nacalai Tesque) antibodies as primary antibodies, and Alexa Fluor 555 Goat anti-rabbit (1:200, Molecular Probes, Eugene, OR) and Alexa Fluor 488 Goat anti-rat (1:200, Molecular Probes) antibodies as secondary antibodies. The stained sections were observed with a confocal laser scanning microscope (FV-300, Olympus, Tokyo, Japan) equipped with objectives (4×, NA 0.16 and 20×, NA 0.75, Olympus).

### *In vivo* imaging

To label blood vessels in the brain, mice were ip-injected with Evans Blue (0.05% in PBS, 4 µl per g of the body weight, 09158-74, Nacalai Tesque). *In vivo* imaging and laser ablation were performed with a custom-built two-photon microscope equipped with a titanium-sapphire pulse laser (Mai Tai DeepSee, Spectra-Physics, Santa Clara, CA) and a water immersion objective (25×, NA 1.05, Olympus). Fluorescence was divided into green and red channels with a dichroic mirror (SDM 570 S, Sigma Koki, Tokyo, Japan), filtered with band-pass filters (500–550 nm (ET525/50 M, Chroma, Bellows Falls, VT) and 590–650 nm (ET620/60 M, Chroma), respectively), and detected with a pair of GAsP-type photomultiplier tubes (PMTs, H7422PA-40, Hamamatsu Photonics, Hamamatsu, Japan). Wave length of excitation laser light was set to either 860 or 920 nm. Full laser power was 15–32 mW (860 nm) and 20–25 mW (920 nm) under the objective, which were adjusted with an acoustic optic modulator (23080-X-0.85-LTD, Gooch & Housego, Melbourne, FL). Craniotomized mice were held with a fixator (Narishige, Tokyo, Japan) placed under the objective. Three-dimensional structures of astrocytes and blood vessels located within a 100 µm depth from the brain surface were acquired by taking image stacks every 1 µm. All the *in vivo* imaging was carried out under the anesthetized condition, and when the imaging procedure lasted for more than 9 h, pentobarbital was additionally injected to the mice. In imaging experiments lasting for more than a day, mice were returned to a cage to recover, and re-injected with another shot before the next observation.

In a subset of experiments, astrocytes were stained with SR101, instead of expressing EGFP, to label astrocytes on the day of craniotomy, and blood vessels were labeled with FITC-dextran instead of EB. Wild type mice were used. SR101 (2.5 µM in PBS, sc-215929, Santa Cruz Biotechnology, Dallas, TX) was applied to the cortical surface after removal of dura, which was washed with washing solution (in mM, 124 NaCl, 2.5 KCl, 1.25 NaH_2_PO_4_, 2 MgCl_2_, 2 CaCl_2_) 5 minutes later before placing a cover glass. And mice were ip-injected with FITC-dextran (4 kDa, 20 mg/ml in PBS, 5 µl per g of the body weight, FD4, Sigma-Aldrich, St Louis, MO).

### Laser ablation

Laser ablation was performed on astrocytic endfeet or blood vessels located within a 100 µm depth from the brain surface. A laser beam was scanned along a line 3–5 µm in length. Laser power was set high (45–115 mW under the objective). The same laser wavelength as used in imaging was used for ablation of astrocytic endfeet. 860 nm was used in ablating vascular walls irrespective of the wave length for imaging because higher power was needed to ablate vascular walls, and 860 nm was more effective in ablation than 920 nm. Ablating irradiation was performed for 0.3–10 s first, and if the ablation was insufficient, further irradiation was repeatedly applied with a higher laser power and longer duration until completion.

### Data analysis

All laser scanning and acquisition control and data analyses were performed with in-house software, TI Workbench, written by T.I. running on a Mac computer^34^. To reduce noise, all acquired images were smoothed with a two-dimensional Gaussian filter. For analysis of three-dimensional structure, image stacks were converted to two-dimensional images with maximum-intensity projection.

## Results

### Astrocytes observed with GFAP-EGFP mice were reactive astrocytes

**Figure 1:**
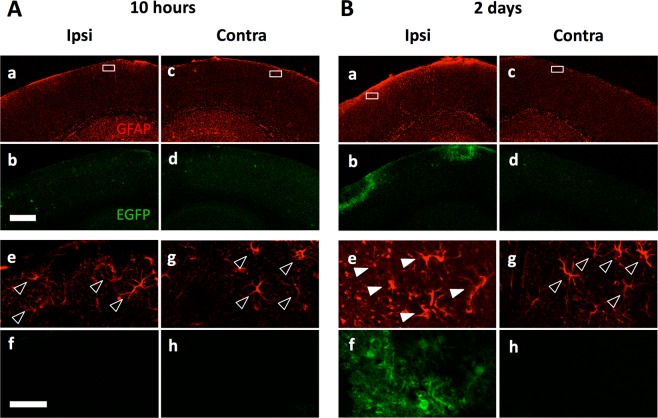
Astrocytes were activated by the cranial window operation on the GFAP-EGFP mice brain. Immunohistochemistry for EGFP (green) and GFAP (red) expression in cerebral cortex 10 hours (**A**) and two days (**B**) after craniotomy in one side (ipsilateral; Ipsi). The contralateral sides (Contra) are shown as a control (Ac,d,g and h). (Ae–h and Be–h) Higher magnifications of the boxes in top panels are shown. GFAP-driven EGFP was only detectable in the operated side of cortex with delay of more than one day. Filled arrowheads and open arrowheads indicate EGFP-positive/GFAP-positive astrocytes and EGFP-negative/GFAP-positive astrocytes, respectively. Scale bar, 500 µm (Ab) and 50 µm (Af).

To confirm the expression of EGFP in the brain of GFAP-EGFP mice, immunohistochemistry was performed for EGFP and GFAP on cerebral cortical sections prepared on the day and two days after the operation for cranial window. The expression level of GFAP was relatively low in operated hemisphere on the day of operation (Fig. 1Aa,e) in the cortical area at about 300 µm depth from the brain surface, which was increased within two days (Fig. 1Ba,e). In control not-operated sides the expression level of GFAP was kept low, comparable to the operated side just after the operation (Fig. 1Bc,d). EGFP expression was not detected unless mice had operation (Fig. 1Ad,h,Bd,h). EGFP expression was obvious only in the operated side two days after (Fig. 1Bb,f) in GFAP-positive astrocytes (Fig. 1Ba,e). The EGFP signal was observed around the operated area, thus the physical vibration and force caused astrocyte activation.

### Astrocytic endfeet re-covered blood vessels after laser ablation

**Figure 2:**
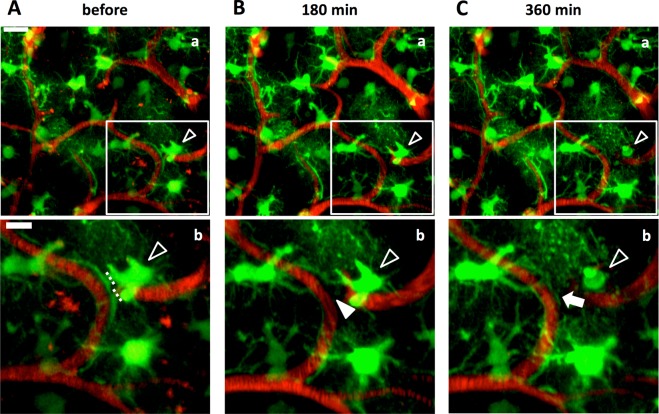
Blood vessels were re-covered by astrocytic endfeet after ablation of endfeet. An astrocytic endfoot covering a blood vessel in the cerebral cortex of a GFAP-EGFP mouse was laser ablated. (**A**) Astrocytes before laser ablation. EGFP-expressing astrocytes (green) covered blood vessels labeled with EB (red). The ablated astrocytic endfoot disappeared from the blood vessel 180 min after the laser irradiation (filled arrowhead in **B**), and the blood vessel was re-covered by another astrocytic endfoot 360 min after (**C**). (Ab, Bb and Cb) Higher magnifications of the boxes in Aa, Ba and Ca, respectively. Dotted lines in Ab indicate the locations of laser ablation. Open arrowheads show the ablated astrocyte, and filled arrowhead and arrow show the stripped blood vessel and a re-covering astrocytic endfoot, respectively. Scale bar, 20 µm (Aa, Ba and Ca) and 10 µm (Ab, Bb and Cb). These images were constructed by maximum intensity z-projection of 20–50 µm depth from the brain surface.

**Figure 3:**
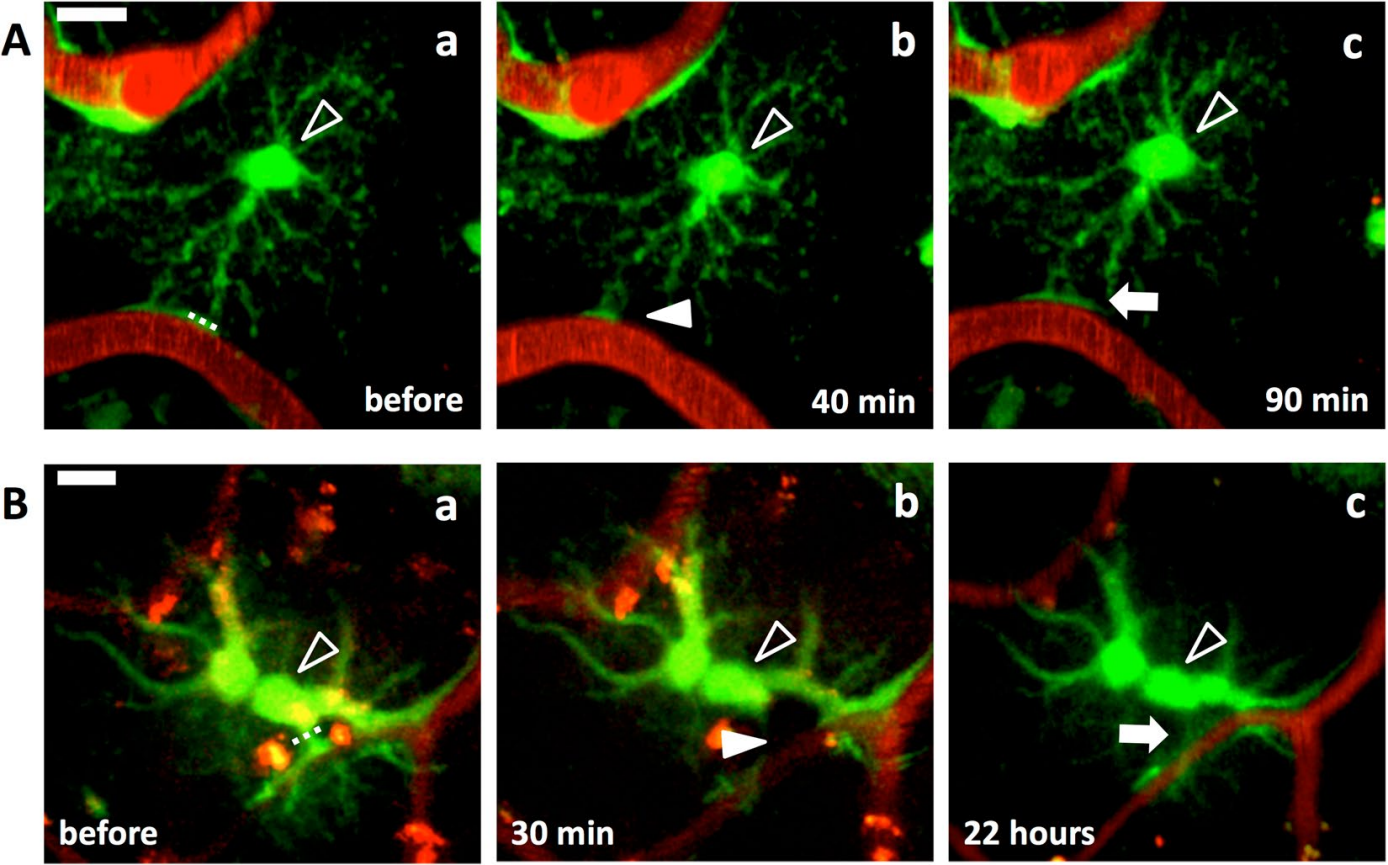
Astrocytic endfeet re-cover blood vessels regardless of life or death of ablated astrocyte and position of the laser ablation. Laser ablation on an endfoot (**A**) and a stalk of process (**B**) of astrocytes. Astrocytic endfoot covering a blood vessel (Aa) disappeared 40 min after the laser ablation (filled arrowhead in Ab), and another endfoot of the same astrocyte re-covered the blood vessel 90 min after (arrow in Ac). (**B**) Laser ablation on a stalk of astrocytic process. The endfoot covering a blood vessel (Ba) disappeared 30 min after the laser ablation (filled arrowhead in Bb) and re-covered the blood vessel 22 h after (arrow in Bc). Open arrowheads point cell bodies of the ablated astrocytes. Dotted line, locations of laser ablation. Scale bar, 10 µm. These images were constructed by maximum intensity z-projections of 30–50 (**A**) and 20–35 µm (**B**) depth from the brain surface.

**Figure 4:**
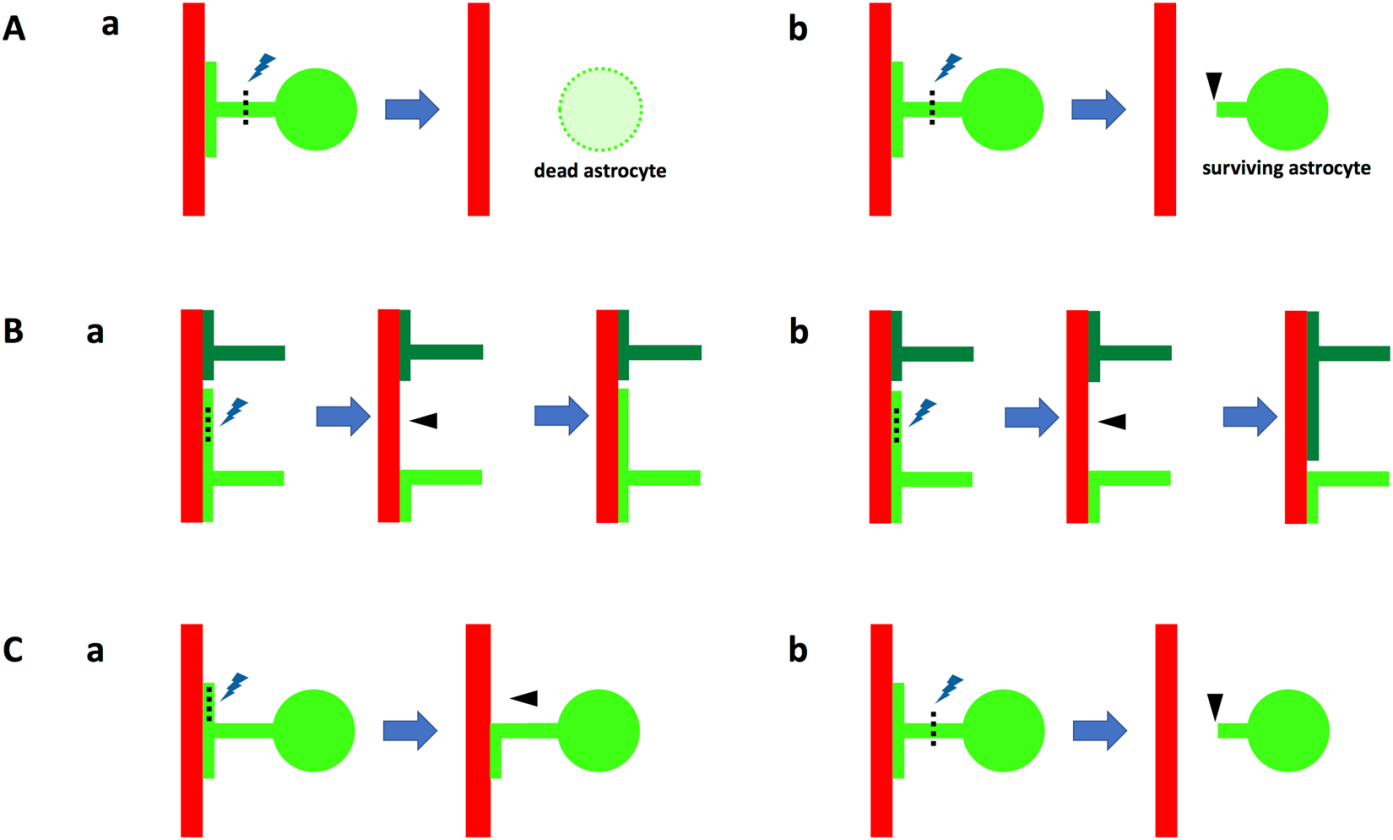
Schematic drawings of laser ablation of astrocytic endfeet showing patterns of astrocytic endfeet behavior after laser ablation. (**A**) Re-covering of stripped blood vessels irrespective of death (Aa) or life (Ab) of ablated astrocytes. (**B**) Stripped blood vessels were re-covered by the endfeet of ablated astrocytes (Ba) or those of other astrocytes (Bb). (**C**) Either endfeet (Ca) or stalks of endfeet (Cb) were laser ablated. Ablated astrocytes are colored in bright green, other astrocytes in dark green and blood vessels in red. Lightning symbols and dotted lines represent laser ablated loci, and arrowheads show removed astrocytic endfeet by laser ablation.

**Figure 5:**
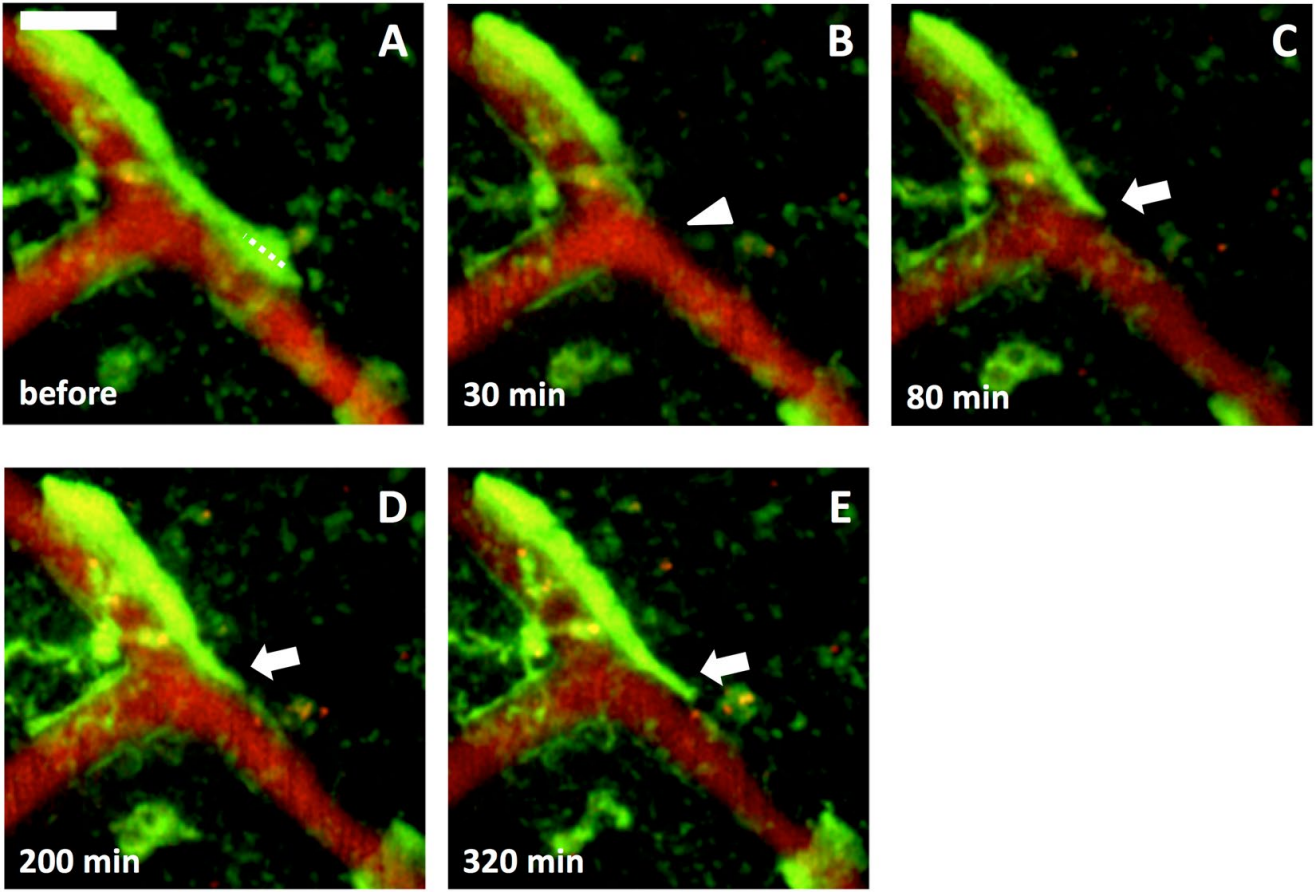
Extension of astrocytic endfeet during re-covering of blood vessels. An astrocytic endfoot covering a blood vessel (**A**) disappeared 30 min after laser ablation (arrowhead in **B**). (**C–E**) The ablated endfoot re-covered the blood vessel by extension (arrows in **C–E**). Dotted line, location of laser ablation. Scale bar, 10 µm. These images were constructed by maximum intensity z-projections of 10–30 µm depth from the brain surface.

To examine the functional relationship between astrocytic endfeet and blood vessels, we destructed only astrocytic endfeet by using the laser ablation method (Materials and Methods) and tracked structural changes of astrocytes in intact brains of GFAP-EGFP mice. Astrocytes expressed EGFP, and blood vessels were labeled with ip-injected EB. Laser ablation of astrocytic endfeet was successful in 54 cases out of 158 trials. In an example shown in Fig. 2, an endfoot detached from a blood vessel just after laser ablation of the stalk of the endfoot, while the cell body of ablated astrocyte and surrounding astrocytes remained intact (Fig. 2A,B). Six hours later the stripped blood vessel was re-covered by an endfoot of another astrocyte, while the ablated astrocyte died (Fig. 2C). Such re-cover of stripped blood vessels by an endfoot was observed in 26 cases out of the 54 cases of successful endfoot ablation within the range from 50 min to 5 days. Whether ablated astrocytes died was irrelevant to the re-covering of stripped blood vessels by an endfoot; ablated astrocytes died but re-coverage appeared in 8 cases (6 mice, Figs 2C and 4Aa), and ablated astrocytes survived and re-coverage appeared in 18 cases (12 mice, Figs 3A,B and 4Ab). Thus, the re-covering endfeet stemmed from either the original ablated astrocytes (n = 9, 8 mice, Figs 3A and 4Ba) or other astrocytes (n = 9, 7 mice, Figs 2 and 4Bb), and in the remaining (n = 8, 5 mice) the origin of the re-covering endfeet could not be identified. In 13 (10 mice) out of the 26 cases of blood vessels re-cover by endfeet, endfeet that had already been touching the stripped blood vessels extended themselves to re-cover stripped blood vessels. In an example shown in Fig. 5, the extension of endfeet started between 30 and 80 min after ablation. In the remaining 13 cases, the origin of astrocytic endfeet was not identified.

Laser ablation was applied at astrocytic endfeet (Fig. 4Ca), stalks of the astrocytic process (Fig. 4Cb) or the cell body of astrocytes (n = 43, 106 and 9, respectively). Among them, endfoot ablation was successful in 19, 32 and 3 cases, respectively, and further re-cover of stripped blood vessels was observed in 13 (10 mice, Fig. 3A), 13 (9 mice, Fig. 3B), and 0 cases, respectively. Astrocytes located far from the laser ablation points maintained their morphology: they did not extend or retract their endfeet during observation periods up to four days (n = 11, data not shown).

### Removal of astrocytic endfoot did not cause BBB breakdown

**Figure 6:**
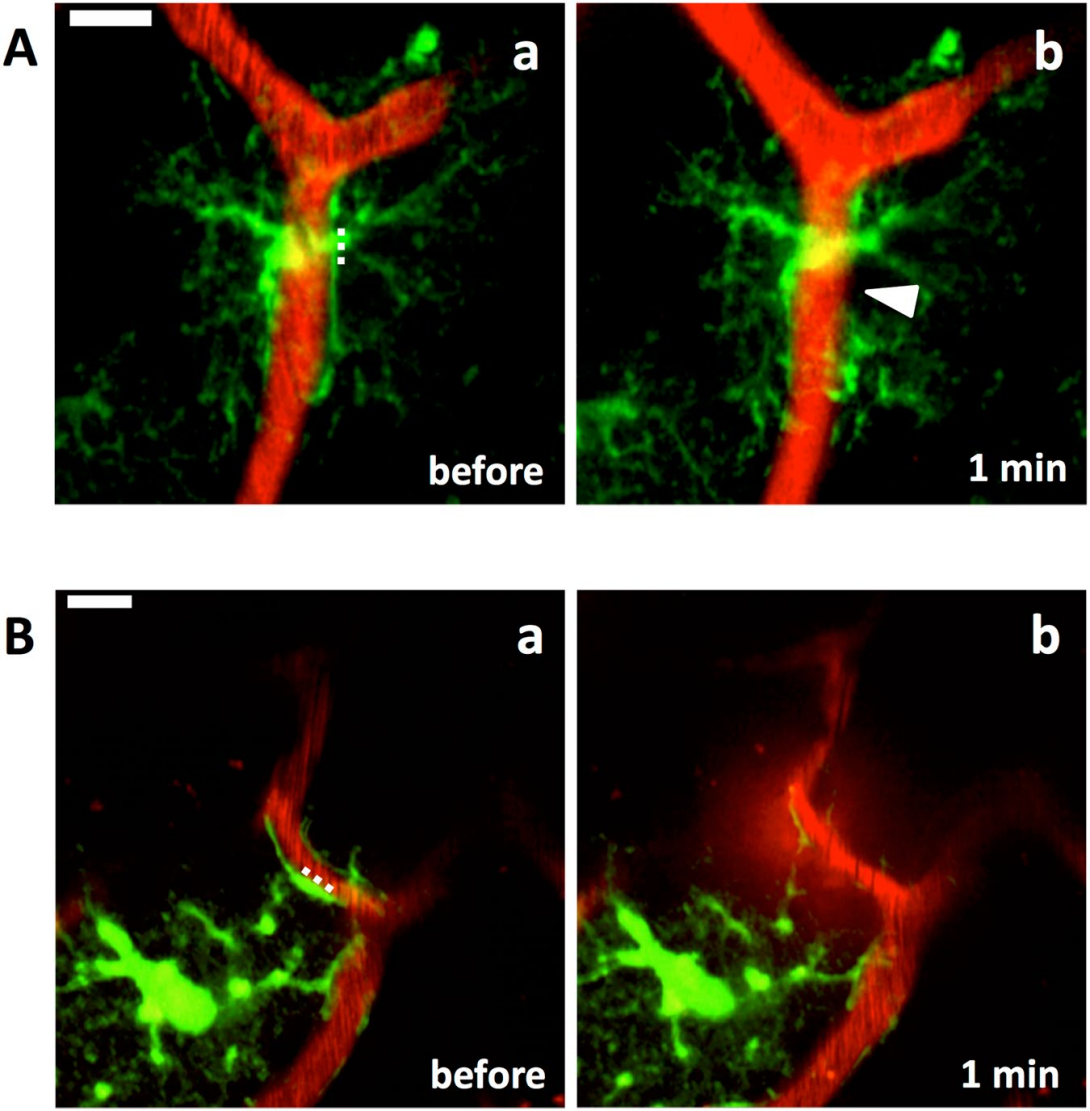
Laser ablation on astrocytic endfeet did not cause BBB breakdown. (**A**) An astrocytic endfoot disappeared (arrowhead in Ab) one min after laser ablation (dotted line). Leakage of EB was not observed. (**B**) Laser ablation on vascular wall (dotted line) caused a leak of EB into the parenchyma one min after the laser ablation (Bb). Scale bar, 10 µm. These images were constructed by maximum intensity z-projections of 50–60 (**A**) and 35–38 µm (**B**) depth from the brain surface, respectively.

Although astrocytes are considered to play a role in maintaining BBB integrity^14,15^, evidence indicating that astrocytic endfeet constitute the physical barrier that prevents non-specific molecular exchange between the inside and outside of blood vessels has not been presented yet. To clarify this, leakage of EB from blood vessels was carefully checked as indication of disruption of BBB by short-interval time lapse imaging. Leakage of EB was not observed when the astrocytic endfoot was ablated (n = 6, 4 mice, Fig. 6A). Conversely, when vascular walls were ablated, leakage of EB was observed (n = 10 out of 25 trials, Fig. 6B); i.e., BBB was physically disrupted. These results indicate that the astrocytic endfoot is not an integral part of the immediate physical blood vessel barrier.

### Not activated astrocytes re-covered blood vessels

**Figure 7:**
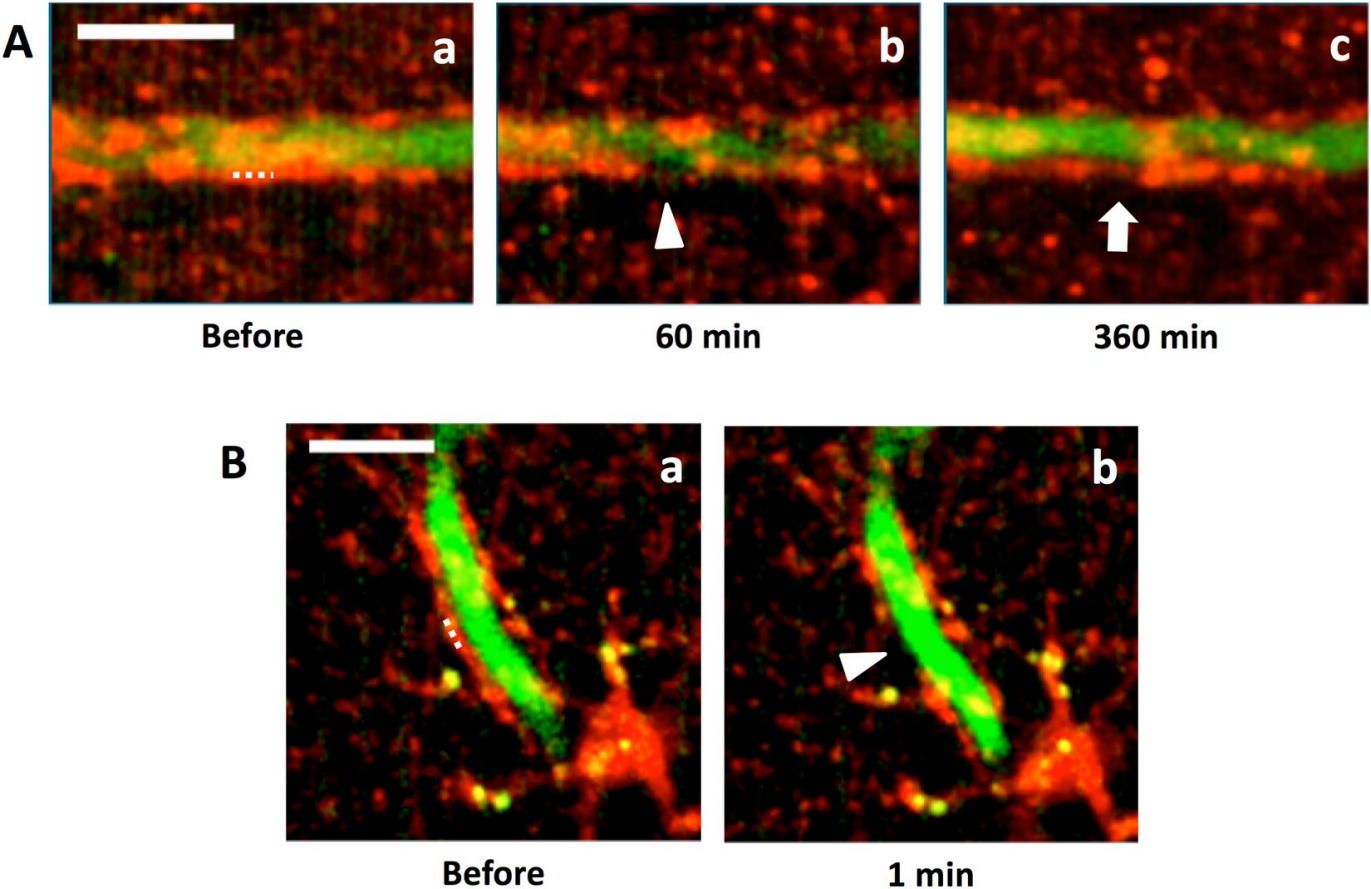
Astrocytes and blood vessels were labeled with SR101 and FITC-dextran. Astrocytes were labeled with SR101 (red) and blood vessels were labeled with FITC-dextran (4 kDa, green). Astrocytic endfeet covering blood vessels were laser ablated. (**A**) The ablated endfoot disappeared 60 min after the laser irradiation (arrowhead in Ab), and the blood vessel was re-covered by an astrocytic endfoot 360 min later (arrow in Ac). Dotted line in Aa indicates the location of laser ablation. (**B**) An astrocytic endfoot disappeared (arrowhead in Bb) one min after laser ablation (dotted line in Ba). Leakage of FITC-dextran was not observed. Scale bars, 10 µm. Images were constructed by maximum intensity z-projections of 60–70 (**A**) and 25–35 µm (**B**) depth from the brain surface.

The dynamic behavior of astrocytic endfoot found in this study was obtained from activated astrocytes. To test whether endfeet of not activated astrocytes also show the re-covering feature after laser ablation, astrocytes were stained with SR101 on the day of craniotomy. Within 10 h after craniotomy endfeet of astrocytes showed re-cover of stripped area along blood vessels (n = 3 out of 4 ablations, 3 mice, Fig. 7A). This result suggests that the re-covering nature of astrocytic endfoot is rather a general feature than specific to active astrocytes. In this series of experiments, blood vessels were labeled with dextran-FITC instead of EB. EB circulates in the blood stream as a protein-bound form, mainly bound with albumin (65–70 kDa), which did not leak out from endfoot-stripped blood vessels. To test if the stripped blood vessels were actually disrupted and permeable to smaller molecules than albumin, dextran-FITC (4 kDa) was used. Leak of dextran-FITC was not observed when the astrocytic endfeet were removed from blood vessels by laser ablation (Fig. 7B, n = 5, 4 mice), indicating that the barrier function of endfoot-stripped blood vessels is not disrupted.

## Discussion

This study is the first to apply the laser ablation method to astrocytic endfeet in a mouse brain using 2PLSM. This method enabled us to reveal that astrocytes actively fill gaps in blood vessel covering created by laser ablation by using astrocytic endfeet, irrespective of whether the filling astrocyte was the target of ablation or not. In addition, the procedure revealed that the astrocytic endfoot is not an integral part of the direct physical barrier.

Previous studies demonstrated that astrocytes interact with blood vessels to regulate blood flow, to be involved in keeping BBB integrity and to supply nutrients from blood to neurons^1,13,35^. We observed re-covering of blood vessels by astrocytic endfeet after laser ablation of astrocytic endfeet covering the blood vessels irrespective of life or death of the ablated astrocytes (Figs 2 and 3) and irrelevant to astrocytic reactivity (Figs 2, 3 and 7), suggesting that an active mechanism that maintains covering of blood vessels by astrocytic endfeet in the brain exists and may be indispensable for the astrocyte-blood vessel interaction. The glymphatic system is a clearance mechanism of interstitial solutes to drain the brain parenchyma, in which the perivascular astrocytic endfeet serve as a sieve^36^. A clearance assay with a radio-tracer revealed that interstitial solute clearance was reduced by ~70% in AQP4-null mice^37^. Additionally, glymphatic dysfunction has been demonstrated in several neurodegenerative disease models^38^. Thus, the covering of blood vessels by astrocytic endfeet may play a fundamental role in sustaining CNS in the physiological condition. The molecular mechanisms for this active maintenance of blood vessels covered by astrocytic endfoot are intriguing: unknown factors released from endothelial cells or perivascular cells such as astrocytes or pericytes might induce extension of astrocytic endfeet on which receptors for the unknown factor are expressed, or microglia could be participating in sensing the rupture of endfoot covering and induction of endfoot extension.

Not all astrocytes express GFAP^39^; this was also affirmed in this study by the observation that GFAP-driven EGFP was expressed in a subpopulation of astrocytes (Fig. 1). Therefore, not all astrocytic endfeet covering blood vessels were labeled with EGFP in the GFAP-EGFP mice. Only 48% of stripped blood vessels were re-covered with astrocytic endfeet within a few days. The remaining 52% of them were not re-covered by fluorescent endfeet even within a longer observation period. Considering the low expression rate of EGFP among astrocytes, many if not all stripped blood vessels may have been re-covered by EGFP-negative astrocytic endfeet.

Although SR101 is widely used to stain astrocytes in *in vivo* experiments, hyper excitation of neurons has been known as its side effect^40,41^. We consider that this side effect might be minute, if any, in this study, because the concentration of SR101 in this study (2.5 µM) was much lower than the suggested threshold (between 50 and 250 µM)^40^, and further, SR101 was not injected into brain parenchyma but applied on the brain surface only for 5 min then washed. And the re-cover feature of astrocytic endfoot after laser ablation was similarly observed in experiments where EGFP was used as an astrocytic marker as well.

Ablation studies of astrocytes by using genetic toxin expression have reported that the integrity of BBB was not disrupted even without covering of blood vessel by astrocytic endfeet, on the basis of immunohistological observations on fixed mouse spinal cords^18,19^. To examine the functional role of astrocytic endfeet in living tissue, we ablated not entire astrocytes but only astrocytic endfeet that covered blood vessels and monitored astrocytes and blood vessels *in vivo* in this study. Breakdown of BBB was not observed (Figs 6A and 7B), which is in good accordance with the previous studies. Only when vascular walls were ablated, EB leaked out to brain parenchyma (Fig. 6B). Thus, we conclude that astrocytes are not essential for the immediate physical barrier of BBB.

The effect of laser ablation seemed confined in the target cellular compartments as is shown in Figs 2A and 7A: the vascular wall structure was not disrupted even when the laser irradiation was focused within a 1 µm distance. Although the optical conditions are different, a laser ablation study on neuronal spines in *in vivo* preparation reported no microglial activation^26^, suggesting that the level of laser power used for ablating subcellular structure in *in vivo* conditions may not be high enough to activate nearby microglia. However, it would be still possible that nearby cells, e.g. microglia, were also activated by the laser. Whether the re-cover of astrocytic endfoot after laser ablation is an autonomous feature of astrocytes or other cell types also play roles is needed to be elucidated in following studies.

Astrocytes are known to become reactive after brain injury or inflammation occurs, and play important roles such as protecting neurons and limiting inflammation^42^. It has been reported that craniotomy induces reactivity in astrocytes^43,44^. We confirmed that on the day of craniotomy astrocytes were not reactive. And these non-reactive astrocytes also re-covered blood vessels after stripped by laser ablation. Thus, it is worth noting that the astrocytic functions observed in this study, namely, re-covering stripped blood vessels is inherent to not only reactive but also non-reactive astrocytes.

## Data Availability

The datasets generated and analyzed during the current study are available from the corresponding authors on reasonable request.
